# Rational development of a combined mRNA vaccine against COVID-19 and influenza

**DOI:** 10.1038/s41541-022-00478-w

**Published:** 2022-07-26

**Authors:** Qing Ye, Mei Wu, Chao Zhou, Xishan Lu, Baoying Huang, Ning Zhang, Hui Zhao, Hang Chi, Xiaojing Zhang, Dandan Ling, Rong-Rong Zhang, Zhuofan Li, Dan Luo, Yi-Jiao Huang, Hong-Ying Qiu, Haifeng Song, Wenjie Tan, Ke Xu, Bo Ying, Cheng-Feng Qin

**Affiliations:** 1 State Key Laboratory of Pathogen and Biosecurity, Beijing Institute of Microbiology and Epidemiology, Beijing, 100071 China; 2 Suzhou Abogen Biosciences Co., Ltd., Suzhou, 215123 China; 3 NHC Key Laboratory of Biosafety, National Institute for Viral Disease Control and Prevention, Chinese Center for Disease Control and Prevention, Beijing, China; 4 State Key Laboratory of Virology, College of Life Sciences, Wuhan University, Wuhan, Hubei 430072 China; 5 Research Unit of Discovery and Tracing of Natural Focus Diseases, Chinese Academy of Medical Sciences, Beijing, 100071 China

**Keywords:** RNA vaccines, Virology

## Abstract

As the world continues to experience the COVID-19 pandemic, seasonal influenza remain a cause of severe morbidity and mortality globally. Worse yet, coinfection with SARS-CoV-2 and influenza A virus (IAV) leads to more severe clinical outcomes. The development of a combined vaccine against both COVID-19 and influenza is thus of high priority. Based on our established lipid nanoparticle (LNP)-encapsulated mRNA vaccine platform, we developed and characterized a novel mRNA vaccine encoding the HA antigen of influenza A (H1N1) virus, termed ARIAV. Then, ARIAV was combined with our COVID-19 mRNA vaccine ARCoV, which encodes the receptor-binding domain (RBD) of the SARS-CoV-2 S protein, to formulate the final combined vaccine, AR-CoV/IAV. Further characterization demonstrated that immunization with two doses of AR-CoV/IAV elicited robust protective antibodies as well as antigen-specific cellular immune responses against SARS-CoV-2 and IAV. More importantly, AR-CoV/IAV immunization protected mice from coinfection with IAV and the SARS-CoV-2 Alpha and Delta variants. Our results highlight the potential of the LNP-mRNA vaccine platform in preventing COVID-19 and influenza, as well as other respiratory diseases.

## Introduction

The worldwide pandemic of coronavirus disease 19 (COVID-19), caused by severe acute respiratory syndrome coronavirus 2 (SARS-CoV-2), has led to a global health crisis. As of 17 December 2021, more than 270 million confirmed cases of COVID-19 with approximately 5.33 million deaths have been reported globally by the World Health Organization^1^. Varying degrees of clinical symptoms have been reported for SARS-CoV-2 infection, ranging from fever, cough, sore throat, mild or severe pneumonia, and acute respiratory distress syndrome^2,3^.

Worse yet, as the cold season approaches, the possibility of a simultaneous epidemic of other respiratory diseases has also raised serious concerns about the potential risk of coinfection with two or more respiratory pathogens^4,5^. Influenza virus is one of the most common pathogens that was found to establish a concurrent infection with SARS-CoV-2^4,6,7^, and multiple clinical cases of coinfection have been reported^8–10^. Both SARS-CoV-2 and influenza virus are mainly transmitted through respiratory droplets and nasal or throat secretions and cause similar clinical manifestations after a respiratory infection. Several recent studies were conducted to explore whether SARS-CoV-2 and influenza virus coinfection leads to more serious diseases, and the results have suggested that influenza virus infection efficiently facilitates SARS-CoV-2 entry into host cells, leading to prolonged pneumonia with more severe lung lesions than single infection^11–13^. Coinfection with SARS-CoV-2 and influenza virus also causes more severe weight loss and a higher proportion of death in mammals^12–15^, likely resulting in a more serious threat especially during the flu season^16^. Therefore, the development of a combined vaccine is urgently needed to reduce the risk of infection with both pathogens.

In the past decade, messenger RNA (mRNA)-based vaccines have been developed as an effective approach to overcome the existing bottleneck of conventional vaccines in the prevention of infectious diseases^17–19^, and lipid nanoparticles (LNPs) have been demonstrated to serve as an effective system for mRNA delivery^20^. During the COVID-19 pandemic, mRNA vaccines developed by Moderna and Pfizer/BioNTech that encode the spike protein of SARS-CoV-2 were authorized for use in humans for the first time^21–23^. In addition, mRNA vaccine candidates against other respiratory viral diseases, including influenza, are also under different stages of development. LNP-delivered mRNA vaccines encoding the hemagglutinin (HA) proteins of H10N8 and H7N9 were demonstrated to elicit a robust immune response with high levels of protective antibodies according to a phase 1 clinical trial^24,25^.

Previously, we reported a lipid nanoparticle-encapsulated mRNA (mRNA-LNP) vaccine (ARCoV) encoding the receptor-binding domain (RBD) of the SARS-CoV-2 spike protein, which is currently being evaluated in multicenter phase 3 clinical trials^26–28^. In the present study, we developed another mRNA vaccine (ARIAV) encoding the HA antigen of influenza A virus (IAV) H1N1. Then, a final combined vaccine formulation named AR-CoV/IAV was designed and prepared as a combination of ARCoV and ARIAV under the same technology platform. Further experiments clearly demonstrated that AR-CoV/IAV immunization readily induces an antigen-specific immune response in mice and confers protection against infection with IAV and SARS-CoV-2 both individually and in combination.

## RESULTS

### Rational design and characterization of an mRNA vaccine candidate (ARIAV) encoding the full-length HA protein of influenza A (H1N1) virus

**Fig. 1:**
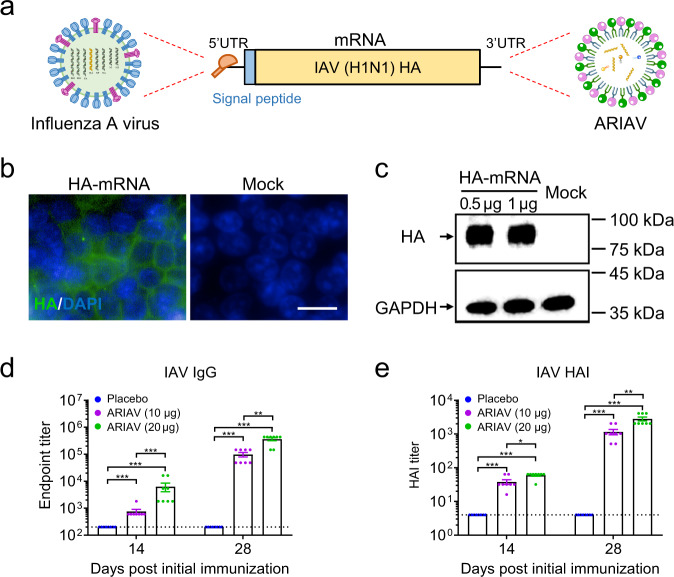
Design and characterization of ARIAV mRNA-LNP encoding HA protein of influenza A (H1N1) virus as a vaccine candidate. **a** Schematic diagram of ARIAV, encoding the full-length HA protein. **b** Indirect immunofluorescence assay of HA protein expression in HEK293T cells 48 h post-transfection. Scale bar, 20 μm. **c** HA expression in HEK293T cells was determined by immunoblotting. **d** HA-specific IgG antibody titers were determined by ELISA. **e** Hemagglutination inhibition (HAI) titers were determined 14 and 28 days post-initial immunization. Data are shown as the mean ± SEM (*n* = 8). Statistical differences were analyzed by using two-tailed unpaired *t* tests. *P < 0.05,**P < 0.01, ***P < 0.001.

We developed an mRNA vaccine candidate (ARIAV) against IAV based on an LNP-encapsulated mRNA platform as previously described (Fig. 1a)^26^. Briefly, we chose the HA of influenza virus A/Wisconsin/588/2019 (H1N1) as the mRNA-encoded antigen; this antigen is included in the WHO recommended composition of quadrivalent or trivalent influenza vaccines for use in the 2021-2022 influenza season in the Northern Hemisphere. We also performed multiple sequence alignment and phylogenetic analysis based on 143 HA gene sequences of influenza A (H1N1) virus isolated in the Northern Hemisphere from 2009 to 2020. A maximum-likelihood tree constructed by the Tamura-Nei model showed that A/Wisconsin/588/2019 clustered closely and exhibited high sequence identity with globally isolated virus strains from 2018 to 2020 (Supplementary Fig. 1).

To evaluate the in vitro expression profile of HA-mRNA, immunofluorescence staining was performed by using a polyclonal HA antibody for H1N1. As expected, a large number of HA-positive cells were detected in mRNA-transfected HEK293T cells, and most fluorescence signals located along the plasma membrane (Fig. 1b). Similarly, western blotting analyses of cell lysates from the mRNA-transfected HEK293T cells also showed a high expression level of HA protein (Fig. 1c; Supplementary Fig. 2a, b). Further flow cytometry assay showed that abundant expression of membrane-bound HA protein (Supplementary Fig. 3 and Supplementary Fig. 4a). To assess the immunogenicity of ARIAV, groups of 6- to 8-week-old mice were immunized with 10 or 20 µg of ARIAV and boosted with the same dose two weeks later; animals injected with placebo served as a control group. Serum samples were collected 14 and 28 days post-initial immunization and subjected to antibody detection. Enzyme-linked immunosorbent assay (ELISA) was performed and demonstrated that ARIAV effectively induced an antigen-specific IgG antibody response (Fig. 1d). The HA-specific functional antibody response was also identified by a hemagglutination inhibition (HAI) assay using influenza virus A/California/07/2009 (H1N1), showing seroconversion in all animals with HAI titers of ~1:38 or 1:60 after one 10 or 20 µg dose of the ARIAV vaccine (Fig. 1e). Booster immunization effectively improved antigen-specific IgG and HAI antibody titers in a dose-dependent manner. The IgG titers reached ~1:364,500, and HAI titers reached ~1:2816 28 days post-initial immunization in the 20 µg group (Fig. 1d, e). The serum IgG and HAI antibodies were below the detection threshold in all of the placebo-vaccinated mice. Our results demonstrate that two immunizations of ARIAV elicit robust antibody responses in mice, supporting its further evaluation as a vaccine candidate against IAV infection.

### A combined mRNA vaccine (AR-CoV/IAV) for COVID-19 and influenza elicits a robust humoral immune response in mice

**Fig. 2:**
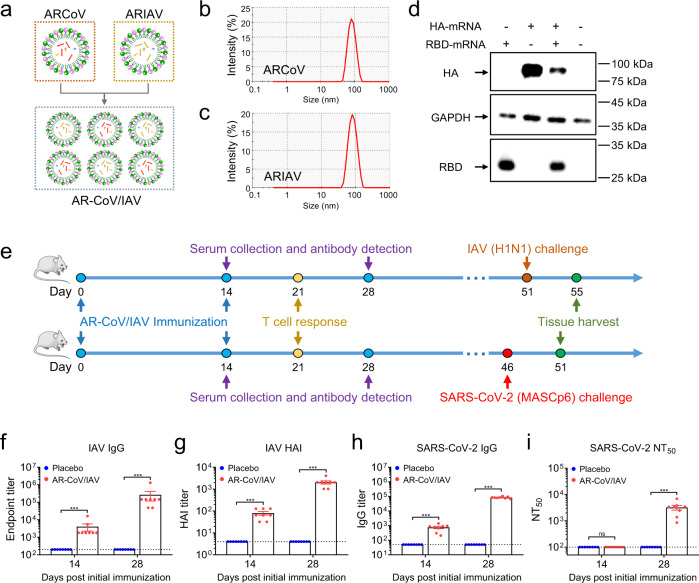
AR-CoV/IAV immunization elicits a humoral immune response in BALB/c mice. **a** Schematic diagram of the AR-CoV/IAV formulation as a combination of ARCoV and ARIAV. **b**, **c** Representative intensity-size graphs of ARCoV and ARIAV. **d** HA and RBD protein expression in HEK293T cells was determined by immunoblotting. **e** Schematic diagram of the experimental design. Groups of mice were immunized with 30 µg of AR-CoV/IAV and boosted with the same dose after two weeks. Serum samples were collected 14 and 28 days post-initial immunization and subjected to antibody detection before an IAV or SARS-CoV-2 challenge. **f**, **h** IAV-HA- and SARS-CoV-2-RBD-specific IgG antibody titers were determined by ELISA. **g** HAI titers were determined 14 and 28 days post-initial immunization. **i** NT_50_ titers against SARS-CoV-2 were determined by using VSV-based pseudovirus. Data are shown as the mean ± SEM (*n* = 8). Statistical differences were analyzed by using two-tailed unpaired *t* tests. ***P < 0.001.

Next, we sought to develop a combined mRNA vaccine candidate by mixing ARCoV and ARIAV under the same LNP-mRNA vaccine platform (Fig. 2a). Particle size measurements indicated that ARCoV and ARIAV exhibited similar average particle sizes of 78.9 and 77.95 nm, respectively, with particle dispersion indices (pdi) less than 0.1 (Fig. 2b, c). Next, the expression of both antigens in HEK293T cells was assessed by immunoblotting following transfection of HA- and RBD-encoded mRNAs, suggesting abundant expression of HA and RBD in cell lysates and supernatants, respectively (Fig. 2d; Supplementary Fig. 2a–c).

Next, as the molecular weight of HA-encoded mRNA is nearly double that of RBD-encoded mRNA, we delivered 30 µg of AR-CoV/IAV as a single vaccination dose that contains 20 µg of HA-encoded mRNA and 10 µg of RBD-encoded mRNA. To evaluate the immunogenicity of AR-CoV/IAV, groups of 6- to 8-week-old mice were immunized with 30 µg of AR-CoV/IAV and boosted with the same dose after two weeks (Fig. 2e). At 14 and 28 days post-initial immunization, ELISA results showed that AR-CoV/IAV vaccination effectively induced the production of IAV-HA and SARS-CoV-2-RBD antigen-specific IgG antibodies (Fig. 2f, h). We also detected high levels of HAI antibodies and of neutralizing antibodies against SARS-CoV-2. The HAI antibodies reached ~1:2048 (Fig. 2g), and the SARS-CoV-2-specific neutralizing antibodies reached ~1:3192 with a 50% neutralization titer (NT_50_) (Fig. 2i) at 28 days post-initial immunization. All of the placebo-vaccinated mice showed undetectable antibody levels for SARS-CoV-2 or IAV. These results suggest that two immunizations with AR-CoV/IAV effectively elicit vaccine-induced protection against SARS-CoV-2 and IAV infection.

### AR-CoV/IAV effectively elicits an antigen-specific T cell immune response

**Fig. 3:**
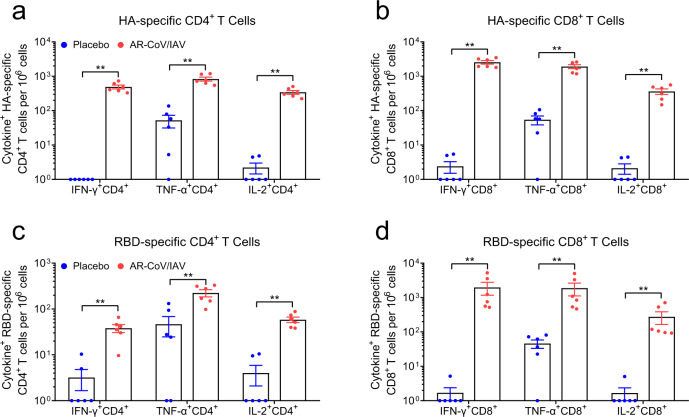
AR-CoV/IAV immunization elicits an antigen-specific T cell immune response in BALB/c mice. **a**, **b** HA-specific CD4^+^ and CD8^+^ T cells producing IFN-γ, TNF-α and IL-2 were determined by flow cytometry. **c**, **d** RBD-specific CD4^+^ and CD8^+^ T cells producing IFN-γ, TNF-α and IL-2 were determined by flow cytometry. Data are shown as the mean ± SEM (*n* = 6). Statistical differences were analyzed by using unpaired *t* tests. **P < 0.01.

We also evaluated the cellular immune response induced by the combined vaccine at 7 days after the second immunization. Flow cytometric analysis was performed to show antigen-specific T cell responses in the spleen of immunized mice, indicating an obvious increase in HA- and RBD-specific polyfunctional CD4^+^ T cells secreting interferon γ (IFN-γ), tumor necrosis factor alpha (TNF-α), or interleukin-2 (IL-2) in AR-CoV/IAV immunized mice compared with placebo vaccination (Fig. 3a, c; Supplementary Fig. 4b). Our results suggested the predominant expression of T-helper-1 (Th1) cytokines in response to ex vivo stimulation with the full-length HA or RBD peptide pool. In addition, HA- and RBD-specific CD8^+^ T cells secreting IFN-γ, TNF-α, and IL-2 were also detected in AR-CoV/IAV-immunized mice (Fig. 3b, d). Moreover, enzyme-linked immunosorbent spot (ELISPOT) analysis revealed significant induction of IFN-γ, TNF-α, and IL-2 in the splenocytes from vaccinated mice (Supplementary Fig. 5a, b). Together, the combined mRNA vaccine AR-CoV/IAV effectively activated obvious antigen-specific CD4^+^ and CD8^+^ T cell responses.

### Vaccine-induced protection from IAV and SARS-CoV-2 infection in mice

**Fig. 4:**
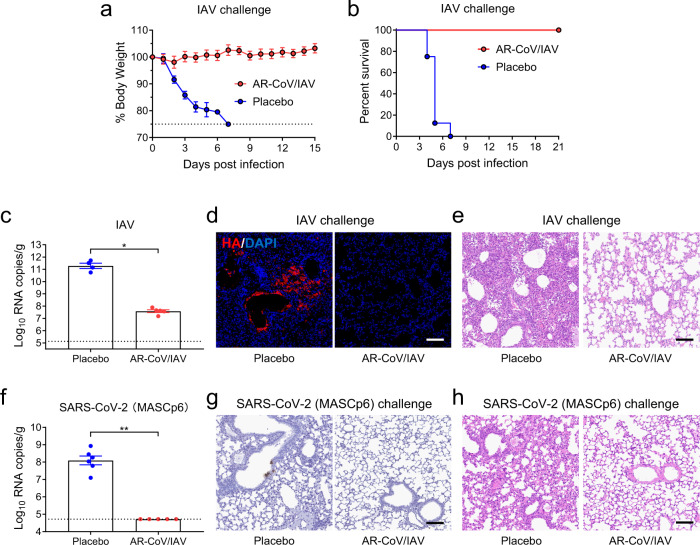
AR-CoV/IAV protects mice from an IAV or SARS-CoV-2 challenge. AR-CoV/IAV- or placebo-immunized mice were challenged with 1.5 × 10^6^ PFU of IAV (A/California/07/2009) (**a–e**) or 1.6 × 10^4^ PFU of SARS-CoV-2 (MASCp6) (**f**–**h**) at the indicated time points post-initial immunization. **a** Weight changes were recorded for 15 days and are shown as the mean ± SEM (AR-CoV/IAV, *n* = 7; placebo, *n* = 8). **b** Mortality was monitored for 21 days after inoculation. **c** Viral RNA copies in the lung tissues of IAV-infected mice (AR-CoV/IAV, *n* = 5; placebo, *n* = 4) were determined by qRT–PCR and are shown as the mean ± SEM. **d** Immunostaining results for HA protein in lung tissues. **e** H&E staining of lung tissues from IAV-infected mice. **f** Viral RNA copies in the lung tissues of SARS-CoV-2-infected mice (AR-CoV/IAV, *n* = 5; placebo, *n* = 6) were determined by qRT–PCR and are shown as the mean ± SEM. **g** ISH assay of lung tissues from SARS-CoV-2-infected mice. **h** H&E staining of lung tissues from SARS-CoV-2-infected mice. Scale bar, 100 μm. Statistical differences between groups were analyzed using two-tailed unpaired *t* tests. *P < 0.05, **P < 0.01.

To explore the protective efficacy induced by AR-CoV/IAV, groups of mice immunized with two doses of AR-CoV/IAV or placebo were challenged intranasally with a lethal dose of 1.5 × 10^6^ PFU of influenza virus A/California/07/2009 (H1N1) 51 days after initial immunization (Fig. 2e). The infected animals were monitored for signs of influenza, and weight changes were also recorded for 15 days post-inoculation. 87.5% of the placebo-immunized mice showed a severe weight loss of more than 20% within 5 days after infection, while the AR-CoV/IAV-immunized mice slowly gained weight, although a slight weight loss was observed only during the first few days after infection (Fig. 4a). In addition, all of the mice that received two immunizations with AR-CoV/IAV survived the lethal dose of IAV infection without symptoms of disease, while the placebo-treated animals died within one week (Fig. 4b). Lung tissues of infected mice were collected and subjected to virological analyses, hematoxylin and eosin (H&E) staining and immunostaining. As expected, abundant viral RNAs of IAV (2.61 × 10^11^ RNA copies/g) were detected in the lung sections of mice treated with placebo 5 days postinfection, whereas a significant decrease in viral RNA loads (4.35 × 10^7^ RNA copies/g) by ~3.8 orders of magnitude was observed in the vaccinated animals (Fig. 4c). An immunofluorescence staining assay also showed robust HA protein expression in lung sections from placebo-treated mice, while viral protein expression was not detected in the vaccinated mice (Fig. 4d). Histopathological analyses revealed a severe pathological change in the lung sections from the placebo group, characterized by alveolar septal thickening, alveolar atrophy, massive inflammatory cell infiltration and vascular congestion. In contrast, the AR-CoV/IAV-vaccinated mice exhibited no typical pathological changes after infection (Fig. 4e). Our results distinctly demonstrate the effective protection of the combined mRNA vaccine against IAV infection.

Next, we utilized a mouse-adapted strain to evaluate the protective efficacy of AR-CoV/IAV against SARS-CoV-2 as previously described^29^. Mice were intranasally challenged with 1.6 × 10^4^ PFU of MASCp6 46 days post-immunization (Fig. 2e). Although high levels of SARS-CoV-2 RNA were detected in the lung tissues from the placebo-immunized group 4 days postinfection, viral RNAs were under the detection threshold among the AR-CoV/IAV-immunized mice (Fig. 4f), indicating potent protection against SARS-CoV-2 infection. Similarly, in situ hybridization (ISH) by RNAscope indicated that viral RNAs could be detected in the lung sections of placebo-treated mice but not in those of the vaccinated group (Fig. 4g). Additionally, AR-CoV/IAV conferred protection to mice from lung injury, as the placebo group developed moderate interstitial pneumonia characterized by thickened alveolar septa and inflammatory cell infiltration, which was not observed in the vaccinated group (Fig. 4h).

### AR-CoV/IAV immunization protects mice from IAV and SARS-CoV-2 variant coinfection

**Fig. 5:**
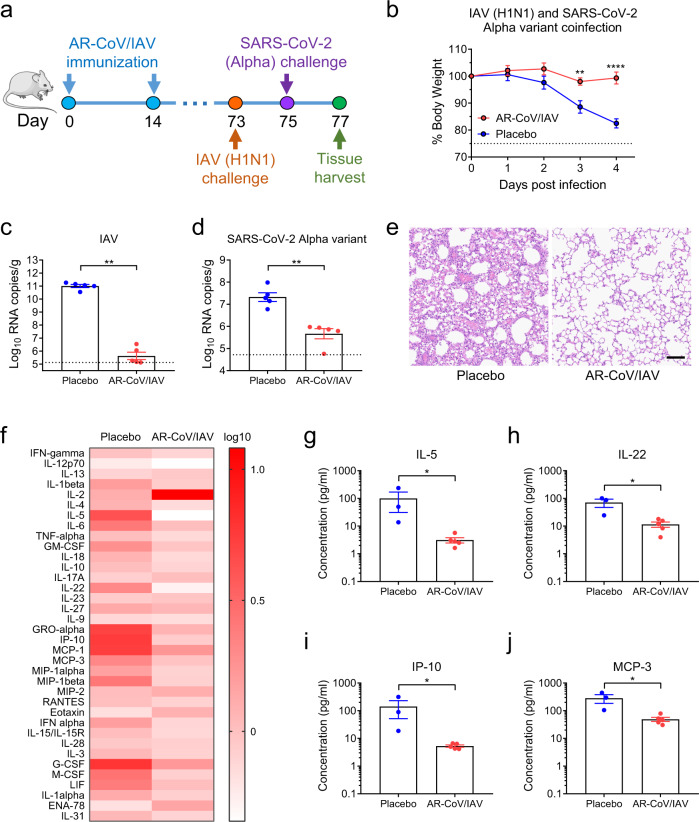
AR-CoV/IAV confers protection against coinfection with IAV and the SARS-CoV-2 Alpha variant in mice. **a** Schematic diagram of experimental design. Groups of AR-CoV/IAV- or placebo-immunized mice were intranasally challenged with 2 × 10^5^ PFU of IAV (A/California/07/2009) 73 days after the initial immunization, followed by infection with 4 × 10^3^ PFU of the SARS-CoV-2 Alpha variant 2 days later. Mice were sacrificed 4 days after IAV infection for viral detection and histopathological analysis. **b** Weight changes in infected mice (*n* = 5) were recorded for 4 days post-infection and are shown as the mean ± SEM. **c**, **d** Viral RNA copies of IAV (**c**) and SARS-CoV-2 (**d**) in the lung tissues of infected mice (*n* = 5) were determined by qRT–PCR and are shown as the mean ± SEM. **e** Histopathologic analysis of lung sections from coinfected mice. Scale bar, 100 μm. **f** Serum cytokine and chemokine analyses were determined by Luminex and are presented as fold changes compared to samples collected before infection (AR-CoV/IAV, *n* = 5; placebo, *n* = 3). **g–j** Concentrations of cytokines and chemokines in serum samples collected post-infection. Data are shown as the mean ± SEM. Statistical differences were analyzed by using two-way ANOVA with multiple comparison tests or two-tailed unpaired *t* tests. *P < 0.05, **P < 0.01, ****P < 0.0001.

**Fig. 6:**
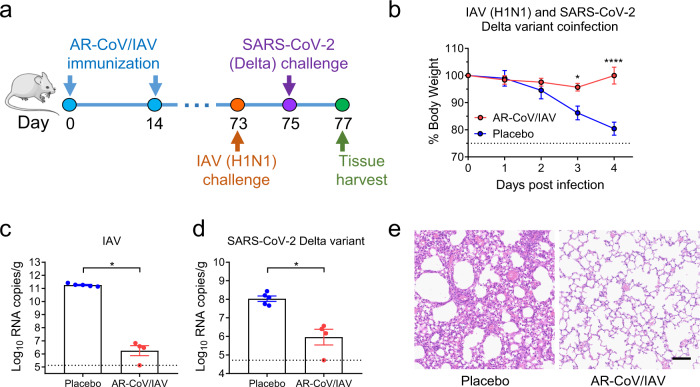
AR-CoV/IAV elicits protection against coinfection with IAV and the SARS-CoV-2 Delta variant in mice. **a** Schematic diagram of experimental design. Groups of AR-CoV/IAV- or placebo-immunized mice were intranasally challenged with 2 × 10^5^ PFU of IAV (A/California/07/2009) 73 days after the initial immunization, followed by infection with 1.2 × 10^4^ PFU of SARS-CoV-2 Delta variant 2 days later. Mice were sacrificed 4 days post-IAV infection for viral detection and histopathological analysis. **b** Weight changes of infected mice (*n* = 5) were recorded for 4 days post-infection and are shown as the mean ± SEM. **c**, **d** Viral RNA copies of IAV (**c**) and SARS-CoV-2 (**d**) in the lung tissues of infected mice (AR-CoV/IAV, *n* = 4; placebo, *n* = 5) were determined by qRT–PCR and are shown as the mean ± SEM. **e** Histopathologic analysis of lung sections from coinfected mice. Scale bar, 100 μm. Data are shown as the mean ± SEM. Statistical differences were analyzed by using two-way ANOVA with multiple comparison tests or two-tailed unpaired *t* tests. *P < 0.05, ****P < 0.0001.

Considering the potential risk of coinfection with different respiratory pathogens during their epidemics, we further determined whether the combined AR-CoV/IAV vaccine confers protection against coinfection with IAV and SARS-CoV-2 variants. We first evaluated the cross-neutralizing antibodies against Alpha and Delta variants prior to coinfection experiments. As expected, mouse sera from AR-CoV/IAV immunized mice are also capable of neutralizing the two variants, and the NT_50_ titers reached ~1:4097 for SARS-CoV-2 Alpha variant and 1:2148 for Delta variant, respectively (Supplementary Fig. 6). Next, we employed a IAV/SARS-CoV-2 coinfection animal model as previously described.^11^ Briefly, groups of mice receiving two immunizations of AR-CoV/IAV or placebo were intranasally inoculated with 2 × 10^5^ PFU of influenza virus A/California/07/2009 (H1N1) 73 days post-initial immunization. Mice were subsequently infected with the SARS-CoV-2 Alpha or Delta variant 2 days later (Figs. 5a and 6a). Infected mice were sacrificed 4 days after IAV infection for viral detection and histopathological analysis. AR-CoV/IAV was demonstrated to protect animals from weight loss; the vaccinated mice experienced only a transient, slight weight loss of less than 5% without typical symptoms of disease, whereas the placebo-treated mice continuously lost weight by more than 15% (Figs. 5b and 6b). Additionally, we observed a significant reduction in the viral RNA levels of IAV and the SARS-CoV-2 variants in the lung tissues from AR-CoV/IAV-vaccinated mice compared with those of the placebo group (Figs. 5c, d and 6c, d). A decrease in influenza virus RNA loads by ~5 orders of magnitude was detected in the vaccinated mice (Fig. 5c and Fig. 6c). For SARS-CoV-2 infection, AR-CoV/IAV showed partial protection against the SARS-CoV-2 Alpha or Delta variant, as the viral RNA loads exhibited 50- and 68-fold decreases compared with those of the placebo group, respectively (Figs. 5d and 6d). Additionally, as previously reported, IAV and SARS-CoV-2 coinfection resulted in an aggravated pathological change in the lung sections of placebo-treated mice, as the histopathological analyses indicated distinct interstitial pneumonia with alveolar septal thickening and infiltration of lymphocytes (Figs. 5e and 6e). In contrast, the vaccinated group showed no obvious signs of inflammation or alveolar lesions. Furthermore, an increased level of cytokine and chemokine expression was detected in serum samples from coinfected mice by Luminex analysis (Fig. 5f). Notably, the vaccinated mice displayed a lower concentration of most cytokines and chemokines after infection (Fig. 5f), including IL-5, IL-22, IP-10 and MCP-3 (Fig. 5f–j), suggesting that the combined mRNA vaccine effectively reduces proinflammatory cytokines and confers protective efficacy against influenza virus and SARS-CoV-2 coinfection.

## Discussion

As the overlap of COVID-19 and influenza epidemics could pose an increased threat of coinfection with different respiratory pathogens, several combination approaches have been reported for the prevention of SARS-CoV-2 and IAV infection. Recently, Bao *et al* demonstrated that a combined vaccination with inactivated COVID-19 vaccine and flu vaccine induced protection against both SARS-CoV-2 and IAV infection^13^. The CRISPR-Cas13 system has also been reported as a specific therapeutic strategy by utilizing CRISPR RNAs targeting the polymerase-encoding genes of influenza virus as well as the replicase and nucleocapsid genes of SARS-CoV-2^30^. Additionally, another group developed a combination of oral bacteria and intranasal inactivated vaccine to protect mice from SARS-CoV-2 and influenza virus infection^31^.

In the current study, we developed a combined mRNA vaccine (AR-CoV/IAV) for COVID-19 and influenza based on the LNP-mRNA platform and reported its immunogenicity and protective efficacy in mice. AR-CoV/IAV comprises the LNP-encapsulated mRNAs encoding IAV-HA and SARS-CoV-2-RBD via a combination of ARCoV and ARIAV (Fig. 2a). The HA protein of the influenza virus was recognized as a promising candidate antigen because of its capacity to elicit HA stalk-specific antibodies with broad reactive responses and protective efficacy against heterologous or heterosubtypic viruses^32–34^. The RBD at the C-terminal region of the SARS-CoV-2 S1 subunit is also a promising vaccine target that has been demonstrated to induce robust neutralizing antibodies and cellular responses.^26^ We noticed that the formulations of ARCoV and ARIAV possess very similar particle sizes (Fig. 2b, c), which provides a distinct advantage for their usage as a combined vaccination. We also verified abundant expression of HA in cell lysates and secreted RBD in supernatants after mRNA cotransfection (Fig. 2d). Accordingly, two doses of AR-CoV/IAV vaccination elicit robust HAI antibodies (>1: 1024) against IAV (Fig. 2g) and neutralizing antibodies (>1: 849) against SARS-CoV-2 (Fig. 2i), that are far beyond the known surrogate correlate of protection^35–37^. Notably, the protective antibody titers were comparable to those from immunization with either ARCoV or ARIAV separately. In addition, our combined vaccine elicited Th1 cytokine-secreting CD4^+^ T cells and IFN-γ^+^ or TNF-α^+^ CD8^+^ T cells (Fig. 3a–d), indicating an increased antiviral capability without causing severe disease^38,39^, which is consistent with the previous studies^26,40,41^. After two immunizations with AR-CoV/IAV, mice were well protected from SARS-CoV-2 and IAV infection or coinfection. Notably, our results demonstrated that the combined vaccine still protected mice from IAV and SARS-CoV-2 variant coinfection over 2 months post-immunization. Although the vaccinated mice were not fully protected, significantly decreased viral loads in lung sections, as well as mild or inconspicuous lung damage were detected without symptoms of either disease, indicating distinct long-term protection against coinfection. Moreover, previous studies have reported a significant elevation of cytokine or chemokine expression in IAV- and SARS-CoV-2-coinfected animals, which may have an intrinsic correlation with inflammatory diseases^12,14^. Immunization with AR-CoV/IAV was shown to contribute to the reduction in numerous proinflammatory cytokines post-coinfection (Fig. 5f–j). Together, our combined vaccine exhibited a potent ability to confer protection against either SARS-CoV-2 or IAV infection.

Due to the constant variation and evolution of respiratory pathogens, including influenza virus and SARS-CoV-2, the development of universal vaccines is important for ensuring vaccine effectiveness against currently circulating strains. Previously, mRNA vaccine platforms were also utilized in the design of universal vaccines to cover various seasonal influenza virus subtypes. Several conserved antigens, including the stalk domain of HA, matrix protein-2 (M2), nucleoprotein (NP) and matrix protein-1 (M1), were considered targets for universal influenza virus vaccines^34,42^. In 2020, a multitargeting mRNA vaccine encoding HA-stalk, NA, M2 and NP was reported to induce robust immune responses in mice with broad protective potential as a universal vaccine candidate^43^. Moreover, as increased genetic diversity has been detected during the rapid evolution of SARS-CoV-2, the development of universal coronavirus vaccines that can induce broad and durable protection against multiple variants is of high priority^44,45^. Combined with the prominent advantages of mRNA technologies, including their intrinsic characteristics for rapid production, excellent safety profile without nuclear entry, and ability to effectively induce humoral and cellular immune responses, the further design of universal vaccines based on mRNA platforms is of great importance to the effective control of COVID-19 and other respiratory disease pandemics in the future.

## Methods

### Cells and Viruses

Vero cells and MDCK cells were maintained at 37 °C under 5% CO_2_ in Dulbecco’s modified Eagle’s medium (DMEM) supplemented with 10% heat-inactivated fetal bovine serum, 10 mM HEPES, and 1% penicillin/streptomycin. The SARS-CoV-2 Alpha variant (CSTR.16698.06.NPRC 2.062100002) was obtained from the Chinese Center for Disease Control and Prevention, and the Delta variant (CSTR.16698.06.NPRC 6.CCPM-B-V-049-2105-6) was obtained from the Chinese Academy of Medical Sciences. The mouse-adapted strain BetaCoV/Beijing/IME-BJ05-P6/2020 (MASCp6) was acquired by serial passaging of a clinical isolate of SARS-CoV-2 in the respiratory tract of BALB/c mice^29^. Influenza virus A/California/07/2009 (H1N1) was grown and titrated in MDCK cells. SARS-CoV-2 variants were titrated on Vero cells. All experiments with infectious SARS-CoV-2 were conducted under biosafety level 3 (BSL3) facilities in the Beijing Institute of Microbiology and Epidemiology.

### mRNA synthesis

Plasmids ABOP-028^26^ and ABOP-140 (GENEWIZ), which encode the RBD region of SARS-CoV-2 (Wuhan-Hu-1, GenBank accession no. MN908947) and the full-length HA of IAV (A/Wisconsin/588/2019, GenBank accession no. MT058823), respectively, were constructed, incorporating the 5’ and 3’ untranslated regions and a poly-A tail. mRNAs were produced in vitro by using a T7 RNA polymerase-mediated transcription from the linearized plasmid. A cap 1 structure was added to the 5’ end of mRNA to maintain mRNA stability and enhance translation efficiency by using Vaccinia Capping Enzyme (Hongene Biotech) and mRNA Cap-2’-O-Methyltransferase (Hongene Biotech). The mRNA products were purified by lithium chloride (LiCl) precipitation.

### LNP Formulation of the mRNA

The mRNA vaccine encoding HA protein of H1N1 was prepared in LNP formulations. Briefly, a lipid mixture including ionizable lipids, 1-,2-distearoyl-sn-glycero-3-phosphocholine (DSPC), cholesterol and PEG-lipid (molar ratios of 50:10:38.5:1.5) was combined with 20 mM citrate buffer (pH 4.0) containing mRNA at a ratio of 1:2 through a T-mixer. The formulation were then diafiltrated in 10 × volume of PBS (pH 7.4), reduced to the desired concentrations through a tangential flow filtration membrane with 100 kD, passed through a 0.22 mm filter, and stored at 2–8 °C until use. All formulations were tested for particle size, distribution, RNA concentration and encapsulation. The combined mRNA vaccine candidate (AR-CoV/IAV) was developed by mixing ARCoV and ARIAV under the same LNP-mRNA vaccine platform. Empty LNPs were utilized as placebo.

### mRNA transfection

HEK293T (ATCC) cells were seeded in 24-well plates at 4 × 10^5^ cells per well and cultured at 37 °C in 5% CO_2_ for 12 h. mRNAs encoding HA or RBD protein were transfected into HEK293T cells using Lipofectamine MessengerMAX Reagent (Thermo Fisher Scientific). Supernatants and cell lysates were harvested at 48 h after transfection. Supernatants were clarified at 13500 × g by centrifugation and then mixed with 5 × SDS loading buffer (nonreducing). Cell lysates were harvested by RIPA lysis buffer (CWBIO) with cOmplete™ Protease Inhibitor Cocktail (Roche), incubated on ice for 30 min, and mixed with 5 × SDS-loading after centrifugation at 13500 × g. The samples were loaded for SDS–PAGE. The secreted RBD protein in supernatants and HA protein in cell lysates were then detected by western blotting using a chimeric MAb for SARS-CoV-2 Spike protein (SinoBiological, 40150-D001, 1:1000) and a rabbit polyclonal antibody for influenza A (H1N1) virus HA protein (GeneTex, GTX127357, 1:1000). GAPDH was detected using GAPDH Loading Control Antibody (Thermo Fisher Scientific, MA5-15738, 1:1000). All blots derive from the same experiment and processed in parallel.

For immunofluorescence staining, HEK293T cells were seeded in 24-well plates at 4 × 10^5^ cells per well and cultured at 37 °C in 5% CO_2_ for 12 h. mRNA encoding HA was transfected into HEK293T cells using Lipofectamine MessengerMAX Reagent (Thermo Fisher Scientific). Transfected cells were fixed with cold methanol/acetone (7:3) 48 h post-transfection and incubated with primary antibody (rabbit polyclonal antibody for H1N1-HA, GeneTex, GTX127357, 1:400) at 37 °C for 1 h. Cells were then washed with PBS three times and then incubated with secondary antibody conjugated to Alexa Fluor 488 (Proteintech, SA00013-2, 1:400). HA-positive cells were examined using a PerkinElmer High Content Analysis System Operetta CLS and processed using Harmony 4.9 software.

### Vaccination and virus challenge experiments

The animal experimental procedure was reviewed and approved by the Animal Experiment Committee of Laboratory Animal Center, AMMS (approval number: IACUC-DWZX-2020-063). Six- to eight-week-old female BALB/c mice were immunized intramuscularly with ARIAV, AR-CoV/IAV or placebo and boosted with an equal dose at 14 days post-initial immunization. 50 μl of mRNA vaccine or placebo were injected into shank muscles. Serum samples were collected prior to immunization and 14 and 28 days after initial immunization and subjected to antibody detection as described below.

Vaccinated mice were anesthetized and infected intranasally with the mouse-adapted strain MASCp6 (1.6 × 10^4^ PFU per mouse) or influenza virus A/California/07/2009 (H1N1) (1.5 × 10^6^ PFU per mouse) at the indicated time points post-immunization. For IAV infection, weight loss was monitored for 15 days, and mortality was monitored for 21 days after inoculation. Animals that lost more than 25% of their initial body weight were humanely anesthetized. Some infected animals (AR-CoV/IAV, *n* = 5; placebo, *n* = 4) were sacrificed 5 days post-challenge for tissue harvest and virological analyses. For SARS-CoV-2 infection, infected animals (AR-CoV/IAV, *n* = 5; placebo, *n* = 6) were sacrificed 4 days post-challenge for tissue harvest and virological analyses.

For the IAV and SARS-CoV-2 coinfection experiments, groups of AR-CoV/IAV-immunized mice (*n* = 5) were intranasally challenged with influenza virus A/California/07/2009 (H1N1) 73 days after initial immunization, followed by infection with a SARS-CoV-2 variant 2 days after IAV infection. Mice were sacrificed 4 days after IAV infection for viral detection and histopathological analysis.

### SARS-CoV-2-specific IgG antibody detection

SARS-CoV-2-RBD-specific IgG titers were determined by ELISA using a commercial kit (Beijing Wantai Biological). Briefly, serum samples were heated at 56 °C for 30 min before use. Inactivated serum samples were serially diluted twofold and added to blocked 96-well plates coated with SARS-CoV-2 RBD. Plates were incubated at 37 °C for 30 min and washed with wash buffer five times. Then, secondary antibody (HRP-conjugated reagent) at a dilution of 1:2 was added to each well to a final volume of 100 µl. Plates were incubated for 30 min at 37 °C and washed five times with wash solution. Fifty microliters of chromogen solution was added, and the solution was incubated for 15 min before being quenched with 25 µl stop solution. Plates were read on a Synergy H1 hybrid multimode microplate reader (BioTek) at 450/630 nm. The endpoint titers were defined according to the manufacturer’s instructions.

### IAV-specific IgG antibody detection

IAV-specific IgG antibody titers were evaluated by ELISA. Flat-bottom, 96-well plates (Corning) were coated with influenza A H1N1 virus HA protein (Sino Biological) at 2 µg/ml to a volume of 50 µl per well. Plates were stored overnight at 4 °C. The following morning, plates were washed five times with PBS with 0.1% Tween 20 (PBST). Then, 100 µl of blocking buffer (PBST containing 5% nonfat milk) was added to each well and incubated for 1 h at 37 °C. Serial threefold dilutions of inactivated serum were added to blocked plates and incubated for 1 h at 37 °C. Plates were then washed with PBST five times, and secondary antibody (horseradish peroxidase-conjugated goat anti-mouse IgG; ZSGB-BIO) at a dilution of 1:5000 was added to each well to a final volume of 100 µl. Plates were incubated for 1 h at 37 °C and washed five times with PBST. Then, 100 µl of chromogen solution was added and incubated for 2 min before being quenched with stop solution. Plates were read on a Synergy H1 hybrid multimode microplate reader (BioTek) at 450/630 nm. The endpoint titers were calculated according to the reciprocal of the highest dilution to achieve an optical density (OD) more than two-fold that of negative serum.

### Pseudovirus-based neutralization assay

Neutralizing antibody titers against SARS-CoV-2 were determined by a pseudovirus-based neutralization assay. Pseudovirus expressing the surface glycoprotein of SARS-CoV-2 (GenBank accession no. QHD43416.1) as well as Alpha variant (GISAID accession no. EPI_ISL_7745829) and Delta variant (GISAID accession no. EPI_ISL_1544070) (Beijing Tiantan Biological Products, 80033, 80043, 80048) were used in the tests. Serial 3-fold dilutions of inactivated serum were incubated with 600 TCID_50_ of pseudovirus in 96-well plates at 37 °C for 1 h, leaving the first and last columns and rows blank to account for edge effects. One hour later, Huh7 cells were added to 96-well plates at 3.5 × 10^4^ cells per well and incubated at 37 °C for 24 h. The supernatants were removed, and luciferase substrate was added to the 96-well plates and incubated for 2 min in darkness. Luciferase activity was determined by using a GloMax 96 Microplate Luminometer (Promega). The 50% neutralization titer (NT_50_) was defined as the serum dilution at which the relative light units (RLUs) were reduced by 50% compared with the virus control wells.

### Hemagglutination Inhibition (HAI) Assay

HA-specific functional antibody titers were determined by HAI assay. Serial 2-fold dilutions of inactivated serum samples were incubated with an equal volume of four agglutinating doses of influenza virus A/California/07/2009 (H1N1) at room temperature. Then, 1% chicken erythrocytes were added to each plate and incubated at room temperature. HAI titers were determined as the reciprocal of the highest serum dilution at which hemagglutination was completely inhibited.

### Enzyme-linked immunospot (ELISPOT) assay

Cellular immune responses were evaluated by using IFN-γ, TNF-α and IL-2 precoated ELISpot kits (MabTech, 3321-4AST, 3511-4APW, 3441-4APW) according to the manufacturer’s instructions. Briefly, single-cell suspensions from mouse spleens were made in complete medium at 3 × 10^6^ cells/ml. A total of 3 × 10^5^ cells (100 µl) per sample were cultured and stimulated using the influenza A virus (A/California/07/2009) HA peptide pool and SARS-CoV-2 RBD peptide pool (peptides are 15mers, with 11 amino acid overlaps, Genscript) at 1.5 µg/ml per peptide. Concanavalin A (ConA, Sigma) was used as a positive control, and RPMI 1640 medium was used as a negative control. Plates were washed with PBS after incubation at 37 °C and 5% CO_2_ for 36 h and incubated with biotinylated anti-mouse IFN-γ, TNF-α and IL-2 antibodies for 2 h at room temperature. Secondary antibody was added to each well to a final volume of 100 μl and incubated for 1 h. Plates were then washed five times with PBS. Then, 100 μl of BCIP/NBT-plus was added and quenched with deionized water. The air-dried plates were read using ImmunoSpot S6 Ultra (CTL). The numbers of spot-forming cells (SFCs) per 1 × 10^6^ cells were calculated.

### Flow Cytometry

To evaluate the expression of membrane-bound HA in vitro, HEK293T cells were transfected with or without 4 μg of HA-encoded mRNA. Cells were collected into 1.5 ml EP tubes after 48 h incubation, and washed twice with 1 mL FACS buffer (PBS with 2% FBS). For membrane staining, 100 μl working solution of the primary antibody to H1N1-HA (Sino Biological, 11055-MM04T, 1:200) was added to each tube to resuspend the cells. The tubes were then placed at 4°C for 30 min in dark. Then, cells were washed two times and incubated in 100 μl working solution of the secondary antibody (Abcam, ab150113, 1:200) at 4 °C for 30 min in dark. The cells were washed twice and resuspended with 200 μl FACS buffer and subjected to flow cytometry.

To evaluate the antigen-specific T cell response, a total of 2 × 10^6^ mouse splenocytes were stimulated with overlapping influenza A virus (A/California/07/2009) HA peptide pool or SARS-CoV-2 RBD peptide pool (peptides are 15mers, with 11 amino acid overlaps, 95% purity, Genscript) at 1.5 µg/ml per peptide at 37 °C with 5% CO_2_. 1 µg/ml anti-mouse CD28 antibody (Biolegend, 102116) and CD49d antibody (Biolegend, 103710) were used to provide co-stimulation. RPMI 1640 medium with DMSO was used as a negative control. After 1 h, Protein Transport Inhibitor (BD Biosciences, 555029, 1:1000) was added to splenocytes and incubated for 8 h. Then cells were collected and washed twice with PBS, blocked with anti-CD16/CD32 antibody (BioLegend, 101303, 1:200) and stained with Zombie Aqua™ Fixable Viability Kit (Biolegend, 423102, 1:200), fluorescently conjugated antibodies to CD3 (BV421, BioLegend, 100341, 1:200), CD4 (FITC, BD, 553046, 1:200), and CD8 (APC/Cyanine7, BioLegend, 100714, 1:200) for 30 min at 4 °C in the dark. Following two washes with PBS, splenocytes were fixed and permeabilized using the Cytofix/Cytoperm kit (BD Biosciences, 554714), and then stained with fluorescently conjugated antibodies to IFN-γ (PE, BioLegend, 505808, 1:200), IL-2 (PE-Cy™7, BioLegend, 503832, 1:200), and TNF-α (PerCP/Cyanine5.5, BioLegend, 506322, 1:200) for 30 min at 4 °C in the dark. Data were collected on FACSVerse flow cytometer (BD Biosciences) and analyzed with FlowJo software.

### RNA extraction and qRT–PCR

Total RNA was isolated using the PureLink™ RNA Mini Kit (Invitrogen) according to the manufacturer’s instructions. SARS-CoV-2 RNA was measured with the following primer-probe set: E_CoV2_F (5’-ACAGGTACGTTAATAGTTAATAGCGT-3’), E_CoV2_R (5’-ATATTGCAGCAGTACGCACACA-3’), and E_CoV2_P (5’-ACACTAGCCATCCTTACTGCGCTTCG-3’). IAV RNA was measured with the following primer-probe set: FluA-F (5’-GACCRATCCTGTCACCTCTGAC-3’), FluA-R (5’-GGGCATTYTGGACAAAKCGTCTACG-3’), and FluA-Probe (5’-TGCAGTCCTCGCTCACTGGGCACG-3’). Amplification was performed using a One Step PrimeScript RT-PCR Kit (TaKaRa, RR064A). PCR was conducted in a LightCycler® 480 Instrument (Roche Diagnostics Ltd).

### RNAscope in situ hybridization

The SARS-CoV-2 RNA ISH assay was performed with RNAscope® 2.5 HD Reagent Kit (Advanced Cell Diagnostics) according to the manufacturer’s instructions. Briefly, 2 μm paraffin-embedded tissue sections were deparaffinized in xylene and rehydrated in a series of graded alcohols. Endogenous peroxidases were quenched with hydrogen peroxide for 10 min at room temperature. Slides were then boiled for 15 min in RNAscope Target Retrieval Reagents and incubated for 30 min in RNAscope Protease Plus before probe hybridization. Tissues were counterstained with Gill’s hematoxylin and imaged with microscopy.

### Histopathological analysis

For histopathology, lung tissues from mice were fixed in 4% neutral-buffered formaldehyde for 48 h, embedded in paraffin, sectioned, and stained with H&E. Sections at 4-μm thickness were examined by light microscopy and analyzed from three independent replicates.

### Multiplex immunofluorescent assay

The HA protein expression in lung tissues was determined by multiplex immunofluorescent assay. Lung paraffin sections were deparaffinized in xylene and rehydrated in a series of graded alcohols. Antigen retrieval was performed in citrate buffer (pH = 6) by heating in a microwave for 20 min at 95 °C followed by a 20 min cool-down period at room temperature. Endogenous peroxidase was quenched with hydrogen peroxide for 20 min, followed by treatment with blocking reagent for 30 min at room temperature. The primary antibody (rabbit polyclonal antibody for H1N1-HA, GeneTex, GTX127357, 1:500) was incubated overnight in a humidified chamber at 4 °C, followed by detection using the Cy3-conjugated secondary antibody (Servicebio, GB21303, 1:300). The slices were imaged using confocal laser scanning microscopy.

### Phylogenetic analysis

A total of 143 HA gene sequences of influenza A H1N1 virus isolated from the Northern Hemisphere from 2009 to 2020 were collected from the influenza virus database of the NCBI. Multiple sequence alignment and phylogenetic analysis were performed by using CLUSTAL W and MEGA 7.0 software. The phylogenetic tree was constructed by the maximum likelihood method.

### Cytokine and chemokines analysis

Cytokines and chemokines expression in serum samples before and after infection were measured using a Mouse Cytokine & Chemokine 36-Plex ProcartaPlex 1 A Panel (Thermo Fisher Scientific) according to the manufacturer’s instructions. The data were collected on Luminex 200 and analyzed by Luminex PONENT (Thermo Fisher Scientific).

### Statistical analysis

Data were analyzed using GraphPad Prism 8 (GraphPad Software). The values shown in the graphs are presented as the mean ± SEM. Statistical differences between groups were analyzed using two-tailed unpaired *t* tests or two-way ANOVA statistical tests, and *P* < 0.05 was considered statistically significant.

### Reporting summary

Further information on research design is available in the Nature Research Reporting Summary linked to this article.

## Supplementary information

Supplementary Information

REPORTING SUMMARY

## Data Availability

The data that support the findings of this study are available from the corresponding author upon reasonable requests.
