# Supplementary material for: Rational development of a combined mRNA vaccine against COVID-19 and influenza
Source: NPJ Vaccines. 2022 Jul 26;7:84. doi: 10.1038/s41541-022-00478-w (PMC9315335; doi:10.1038/s41541-022-00478-w)
Supplement: Supplementary file 1 — Supplementary Information [file 41541_2022_478_MOESM1_ESM.pdf]

Supplementary Materials for

Rational development of a combined mRNA vaccine against COVID-19 and influenza

Qing Ye[1,†], Mei Wu[1,†], Chao Zhou[1,†], Xishan Lu[†2], Ning Zhang[1], Hui Zhao[1], Hang Chi[1], Xiaojing Zhang[2], Dandan Ling[2], Rong-Rong Zhang[1], Zhuofan Li[2], Dan Luo[1], Yi-Jiao Huang[1], Hong-Ying Qiu[1], Haifeng Song[2], Ke Xu[3], Bo Ying[2], Cheng-Feng Qin[1,4*]

*Correspondence: qincf@bmi.ac.cn (C.F.Q)

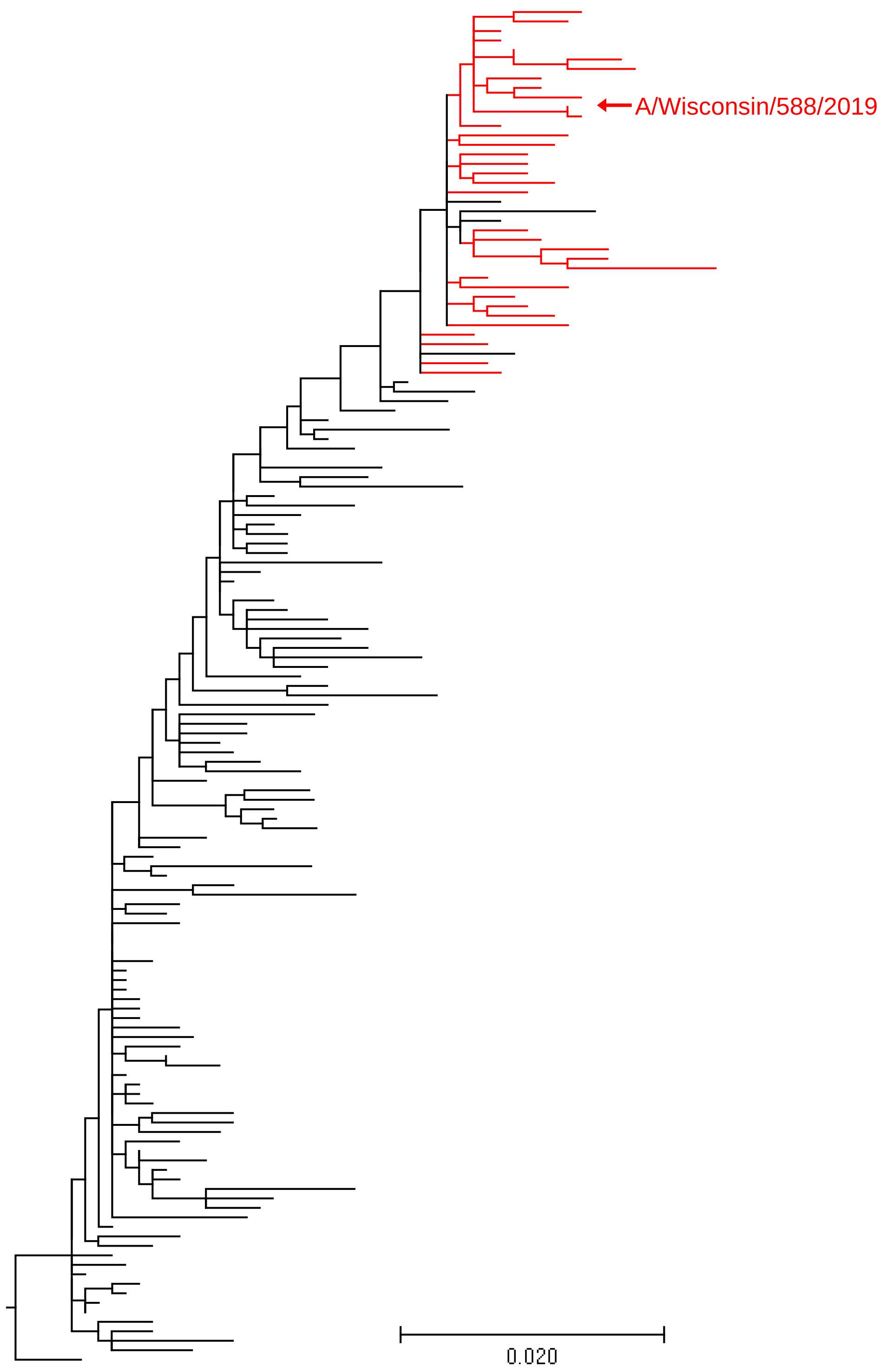

Supplementary Figure 1. A phylogenetic tree was constructed by a maximum likelihood method based on the nucleotide sequence of the HA gene of influenza A (H1N1) virus. The circulating strains isolated from 2018 to 2020 in the Northern Hemisphere are colored red.

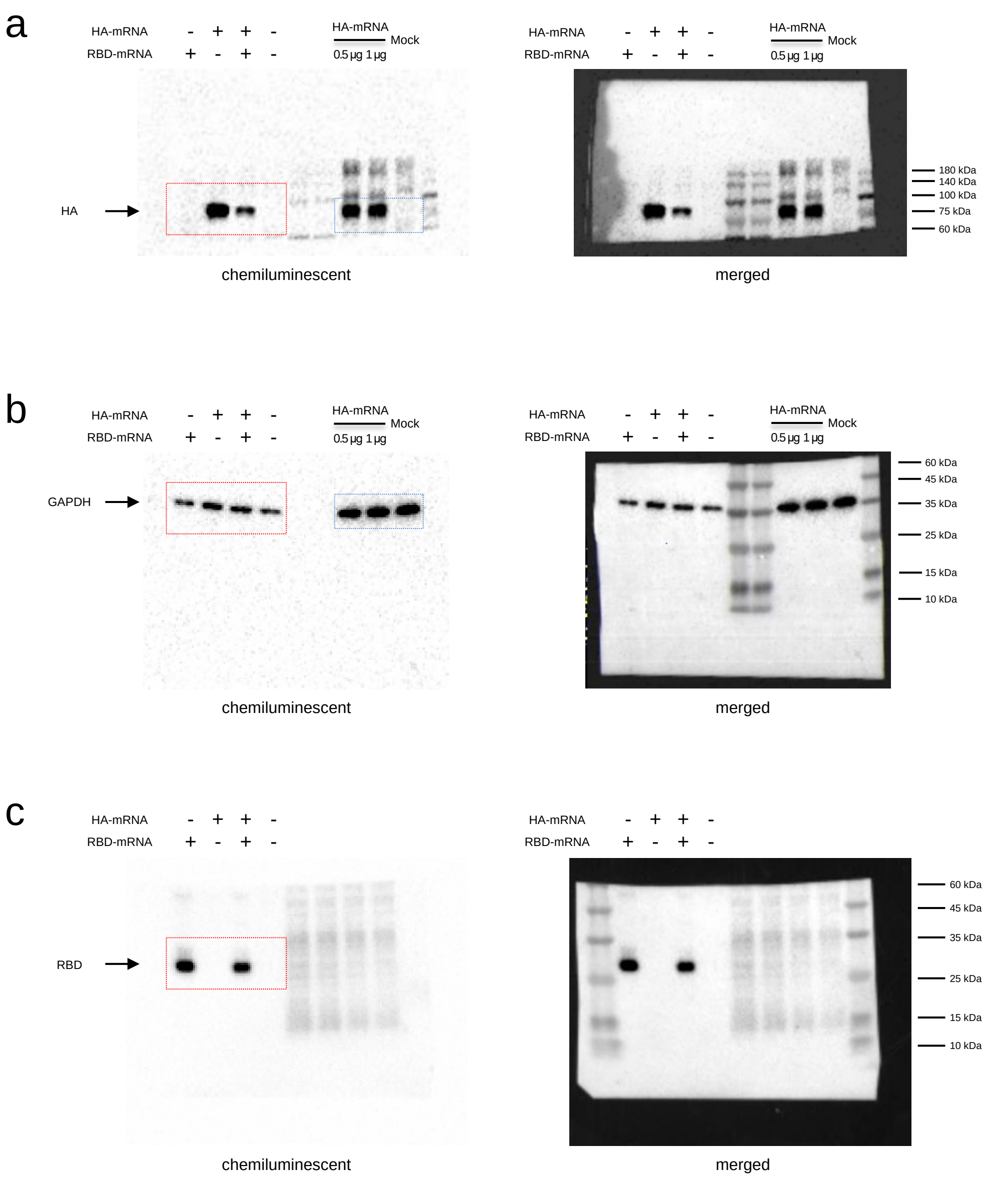

Supplementary Figure 2. Antigen expression was detected by immunoblotting. HEK293T cells were transfected with HA-encoded or RBD-encoded mRNAs. Supernatants and cell lysates were harvested at 48 h after transfection. The presence of HA (in cell lysates) (a), GAPDH (in cell lysates) (b) and RBD (in supernatants) (c) were analyzed by immunoblotting as shown in Fig. 1c and Fig. 2d. All blots derive from the same experiment and processed in parallel.

.

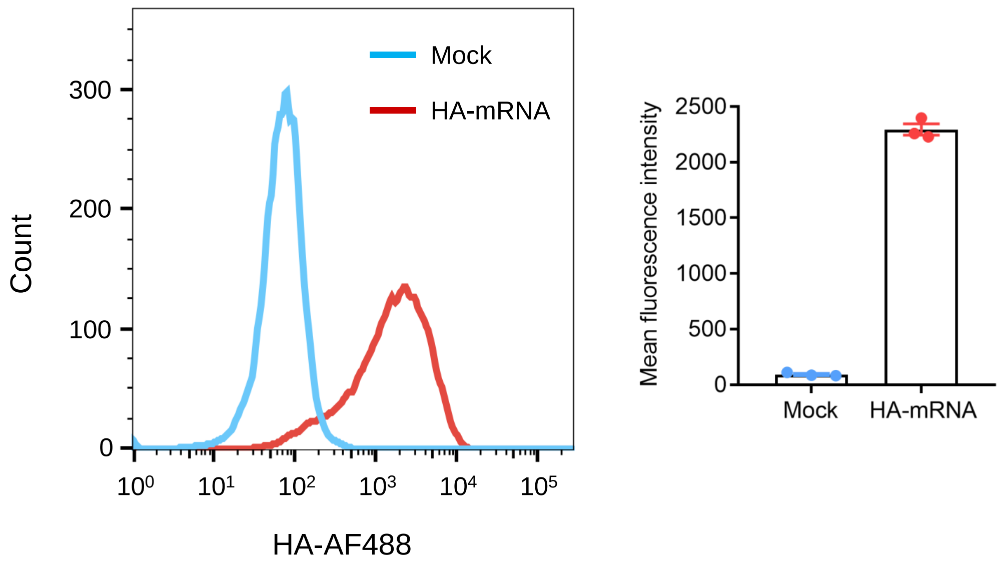

Supplementary Figure 3. In vitro expression of membrane-bound HA was determined by flow cytometry. HEK293T cells were transfected with 4 μg of HA-encoded mRNA for 48 h. Cells were collected, stained with antibody for H1N1-HA, and analyzed by flow cytometry. The data are representative from three independent replicates.

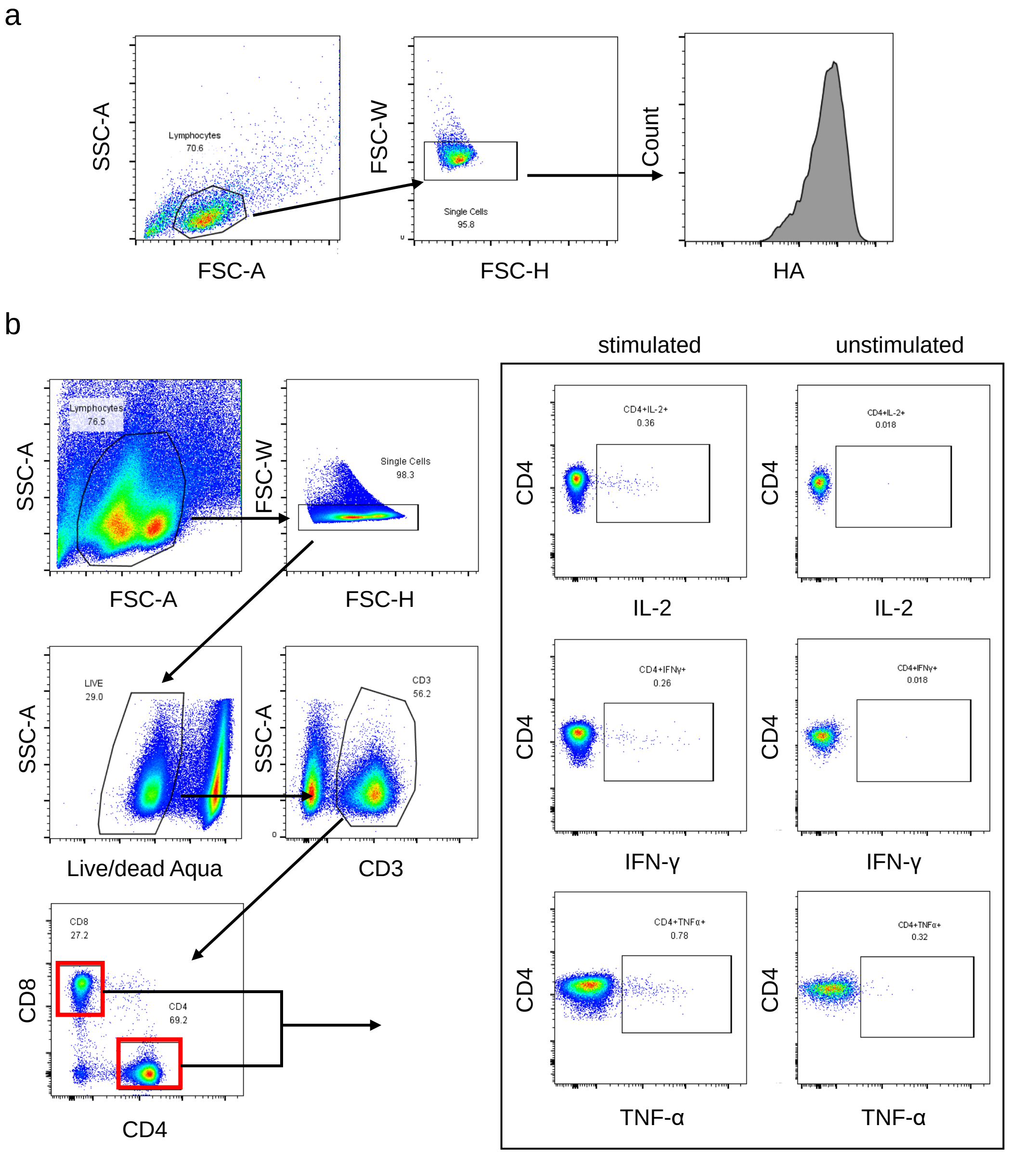

Supplementary Figure 4. Gating strategies for H1N1-HA staining and intracellular cytokine staining. a Gating strategy for HA expression in HEK293T cells presented on Supplementary Fig. 2. **b** Flow cytometric gating strategy for the investigation of T cell responses in AR-CoV/IAV immunized mice presented on Fig. 3a-d.

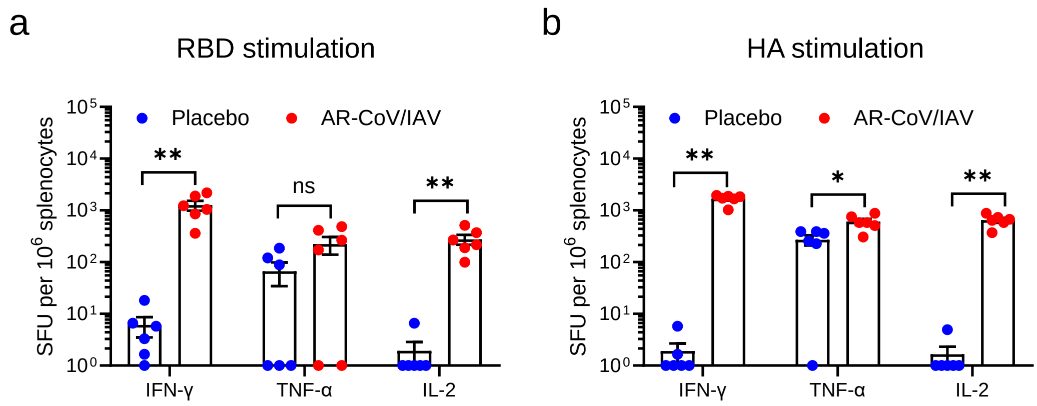

Supplementary Figure 5. AR-CoV/IAV immunization elicits an antigen-specific T cell immune response in BALB/c mice. Production of IFN-γ, TNF-α or IL-2 in splenocytes stimulated with the RBD (a) or HA (b) peptide pool was determined by ELISPOT assay. Statistical differences were analyzed by using two-tailed unpaired t tests. $*P < 0.05$, $**P<0.01$.

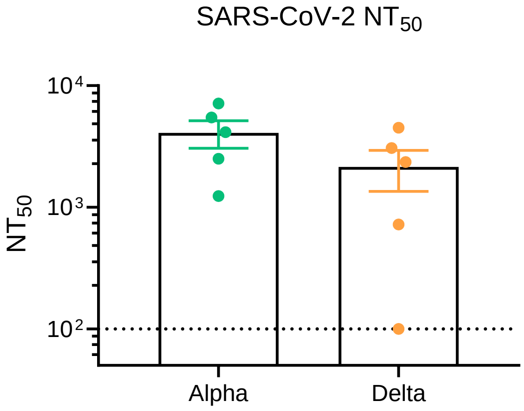

Supplementary Figure 6. AR-CoV/IAV immunization elicits neutralizing antibodies against SARS-CoV-2 variants. NT_{50} titers against SARS-CoV-2 Alpha and Delta variant were determined 73 days post-initial immunization by using VSV-based pseudovirus.
