# Supplementary material for: Rational development of a combined mRNA vaccine against COVID-19 and influenza
Source: NPJ Vaccines. 2022 Jul 26;7:84. doi: 10.1038/s41541-022-00478-w (PMC9315335; doi:10.1038/s41541-022-00478-w)
Supplement: Supplementary file 2 — REPORTING SUMMARY [file 41541_2022_478_MOESM2_ESM.pdf]

nature research

Corresponding author(s): Cheng-Feng Qin

Last updated by author(s): Mar 19, 2022

Reporting Summary

Nature Research wishes to improve the reproducibility of the work that we publish. This form provides structure for consistency and transparency in reporting. For further information on Nature Research policies, see our Editorial Policies and the Editorial Policy Checklist.

Statistics

For all statistical analyses, confirm that the following items are present in the figure legend, table legend, main text, or Methods section.

n/a	Confirmed	
☐	☒	The exact sample size (*n*) for each experimental group/condition, given as a discrete number and unit of measurement
☐	☒	A statement on whether measurements were taken from distinct samples or whether the same sample was measured repeatedly
☐	☒	The statistical test(s) used AND whether they are one- or two-sided<br>*Only common tests should be described solely by name; describe more complex techniques in the Methods section.*
☒	☐	A description of all covariates tested
☒	☐	A description of any assumptions or corrections, such as tests of normality and adjustment for multiple comparisons
☒	☐	A full description of the statistical parameters including central tendency (e.g. means) or other basic estimates (e.g. regression coefficient) AND variation (e.g. standard deviation) or associated estimates of uncertainty (e.g. confidence intervals)
☒	☐	For null hypothesis testing, the test statistic (e.g. *F*, *t*, *r*) with confidence intervals, effect sizes, degrees of freedom and *P* value noted<br>*Give P values as exact values whenever suitable.*
☒	☐	For Bayesian analysis, information on the choice of priors and Markov chain Monte Carlo settings
☒	☐	For hierarchical and complex designs, identification of the appropriate level for tests and full reporting of outcomes
☒	☐	Estimates of effect sizes (e.g. Cohen's *d*, Pearson's *r*), indicating how they were calculated

Our web collection on statistics for biologists contains articles on many of the points above.

Software and code

Policy information about availability of computer code

Data collection	The details of data collection were described in Methods.
Data analysis	The details of data collection were described in Methods.

For manuscripts utilizing custom algorithms or software that are central to the research but not yet described in published literature, software must be made available to editors and reviewers. We strongly encourage code deposition in a community repository (e.g. GitHub). See the Nature Research guidelines for submitting code & software for further information.

Data

Policy information about availability of data

All manuscripts must include a data availability statement. This statement should provide the following information, where applicable:

- Accession codes, unique identifiers, or web links for publicly available datasets
- A list of figures that have associated raw data
- A description of any restrictions on data availability

The data supporting the findings in the manuscript are available under reasonable requests.

April 2020

Field-specific reporting

Please select the one below that is the best fit for your research. If you are not sure, read the appropriate sections before making your selection.

☒ Life sciences ☐ Behavioural & social sciences ☐ Ecological, evolutionary & environmental sciences

For a reference copy of the document with all sections, see nature.com/documents/nr-reporting-summary-flat.pdf

Life sciences study design

All studies must disclose on these points even when the disclosure is negative.

Sample size	Sample size was estimated on the basis of similar research reported in the literature.
Data exclusions	No data were excluded.
Replication	Data were performed with technical replications.
Randomization	Randomization was performed while assigning the animals into experimental groups.
Blinding	The investigators were not completely blinded during the experiment.

Reporting for specific materials, systems and methods

We require information from authors about some types of materials, experimental systems and methods used in many studies. Here, indicate whether each material, system or method listed is relevant to your study. If you are not sure if a list item applies to your research, read the appropriate section before selecting a response.

Materials & experimental systems

n/a	Involved in the study	
☐	☒	Antibodies
☐	☒	Eukaryotic cell lines
☒	☐	Palaeontology and archaeology
☐	☒	Animals and other organisms
☒	☐	Human research participants
☒	☐	Clinical data
☒	☐	Dual use research of concern

Methods

n/a	Involved in the study	
☒	☐	ChIP-seq
☐	☒	Flow cytometry
☒	☐	MRI-based neuroimaging

Antibodies

Antibodies used	The details of antibodies used in the manuscript were described in Methods.
Validation	Validation data about the antibodies obtained from commercial sources are available on the manufacturer's website and/or data sheet.

Eukaryotic cell lines

Policy information about cell lines

Cell line source(s)	MDCK, Vero and HEK293T cells were obtained commercially from ATCC (ATCC, CRL-11268); Huh7 cells were obtained from JCRB (JCRB, 0403)
Authentication	Cells were authenticated according to the features described by ATCC.
Mycoplasma contamination	We confirmed that all cell lines were tested negative for mycoplasma contamination.
Commonly misidentified lines (See ICLAC register)	None.

Animals and other organisms

Policy information about studies involving animals; ARRIVE guidelines recommended for reporting animal research

Laboratory animals	BALB/c, Female, 6-8 weeks.
Wild animals	None.
Field-collected samples	The detail were described in Methods.
Ethics oversight	The Animal Experiment Committee of Laboratory Animal Center, Beijing Institute of Microbiology and Epidemiology (Approval number: IACUC-DWZX-2020-063)

Note that full information on the approval of the study protocol must also be provided in the manuscript.

Flow Cytometry

Plots

Confirm that:

☒ The axis labels state the marker and fluorochrome used (e.g. CD4-FITC).

☒ The axis scales are clearly visible. Include numbers along axes only for bottom left plot of group (a 'group' is an analysis of identical markers).

☒ All plots are contour plots with outliers or pseudocolor plots.

☒ A numerical value for number of cells or percentage (with statistics) is provided.

Methodology

Sample preparation	1. Intracellular cytokine staining assay: Whole spleen were harvested at 21 days post initial immunization. Briefly, mononuclear cells were collected by grinding the spleen with a syringe in the cell strainer, using 10 mL of PBS to pass through the strainer to wash the cells, and the RBCs were lysed with ammonium chloride lysis buffer. Cells were plated at 2 x 10^6 cells/well in 48-well plates and restimulated with overlapping influenza A virus (A/California/07/2009) HA peptide pool or SARS-CoV-2 RBD peptide pool (peptides are 15mers, with 11 amino acid overlaps, 95% purity, Genscript) at 1.5 μg/ml per peptide in the presence of 1 μg/ml of anti-CD28 (BioLegend) and anti-CD49d (BioLegend) at 37°C with 5% CO2. After 1 h, Protein Transport Inhibitor (BD) was added to splenocytes and incubated for 8 h. Controls were treated with DMSO-containing medium. Then cells were collected and washed twice with PBS, blocked with anti-CD16/CD32 antibody (BioLegend) and stained with Zombie Aqua™ Fixable Viability Kit (Biolegend), fluorescently conjugated antibodies to CD3 (BV421, BioLegend), CD4 (FITC, BD), and CD8 (APC/Cyanine7, BioLegend) for 30 min at 4°C in the dark. Following two washes with PBS, splenocytes were fixed and permeabilized using the Cytofix/Cytoperm kit (BD), and then stained with fluorescently conjugated antibodies to IFN-γ (PE, BioLegend), IL-2 (PE-Cy™7, BioLegend), and TNF-α (PerCP/Cyanine5.5, BioLegend) for 30 min at 4°C in the dark. Data were collected on FACSVerse flow cytometer (BD Biosciences) and analyzed with FlowJo software.<br>2. Expression of membrane-bound HA: HEK293T cells were transfected with 4 μg of HA-encoded mRNA and incubated for 48 hours. Cells were collected and washed twice with PBS, then incubated with primary antibody to H1N1-HA (Sino Biological) at 4°C for 30 min in dark. Then, cells were washed two times and incubated in 100 μl working solution of secondary antibody (Abcam) at 4°C for 30 min in dark. The cells were washed twice and resuspended with 200 μl FACS buffer and subjected to flow cytometry. Data were collected on FACSVerse flow cytometer (BD Biosciences) and analyzed with FlowJo software.
Instrument	BD FACSVerse
Software	Flow cytometry data were acquired using BD FACSuite v1.3 and analyzed using FlowJo (Treestar) v10.
Cell population abundance	Cells were not sorted
Gating strategy	Gating strategies are shown in extended data figure

☒ Tick this box to confirm that a figure exemplifying the gating strategy is provided in the Supplementary Information.
